# Fibrils of Truncated Pyroglutamyl-Modified Aβ Peptide Exhibit a Similar Structure as Wildtype Mature Aβ Fibrils

**DOI:** 10.1038/srep33531

**Published:** 2016-09-21

**Authors:** Holger A. Scheidt, Juliane Adler, Martin Krueger, Daniel Huster

**Affiliations:** 1 Institute for Medical Physics and Biophysics, Leipzig University Härtelstr. 16-18, D-04107 Leipzig, Germany; 2 Institute of Anatomy, Leipzig University Eilenburger Str. 14-15, 04317 Leipzig, Germany

## Abstract

Fibrillation of differently modified amyloid β peptides and deposition as senile plaques are hallmarks of Alzheimer’s disease. N-terminally truncated variants, where the glutamate residue 3 is converted into cyclic pyroglutamate (pGlu), form particularly toxic aggregates. We compare the molecular structure and dynamics of fibrils grown from wildtype Aβ(1–40) and pGlu_3_-Aβ(3–40) on the single amino acid level. Thioflavin T fluorescence, electron microscopy, and X-ray diffraction reveal the general morphology of the amyloid fibrils. We found good agreement between the ^13^C and ^15^N NMR chemical shifts indicative for a similar secondary structure of both fibrils. A well-known interresidual contact between the two β-strands of the Aβ fibrils could be confirmed by the detection of interresidual cross peaks in a ^13^C-^13^C NMR correlation spectrum between the side chains of Phe 19 and Leu 34. Small differences in the molecular dynamics of residues in the proximity to the pyroglutamyl-modified N-terminus were observed as measured by DIPSHIFT order parameter experiments.

Fibrillation of amyloid β (Aβ) peptides of different length and degrees of modification and brain deposition as senile plaques represent a hallmark of Alzheimer’s disease. The N-terminally truncated variant, which forms a cyclic pyroglutamate residue at position 3, plays a major role in the development of the disease by forming very toxic aggregates^1^,^2^. For pyroglutamated Aβ variants (pGlu-Aβ) increased oligomerization^3^,^4^,^5^, enhanced fibrillation^6^,^7^,^8^, and increased lipid peroxidation accompanied with a loss of plasma membrane integrity^6^ were reported. These properties can probably explain the enhanced cytotoxic effects of pGlu-Aβ^1^,^4^,^9^. Indeed, pharmacological inhibition of the enzyme that catalyzes the production of the pGlu lactam ring (see Fig. 1) has been reported to reduce the deposition of amyloid plaques and retards the memory decline in mice^10^.

The molecular structure of pGlu-Aβ(3–40) has only been studied for peptides dissolved in TFE-containing buffers by solution NMR^8^,^11^,^12^, or in aqueous media by CD- and FTIR spectroscopy^6^,^7^,^8^,^13^. Furthermore, H/D exchange was monitored by solution NMR^6^. Compared to unmodified Aβ(1–40) or Aβ(1–42), some studies claimed a higher propensity of pGlu-Aβ to from β-sheet structure^7^,^12^, while another paper reported a higher α-helical content and a decreased β-sheet propensity^13^.

A direct comparison of the molecular structure on the amino acid level in the fibrillar state of pGlu-Aβ(3–40) and wildtype (WT) Aβ fibrils is missing. For the study of the molecular structure of fibrils, solid-state NMR spectroscopy is the method of choice^14^ to provide details of the molecular structure of amyloid fibrils in different stages of fibrillation. Overall and in relatively good agreement, the models have identified two β-strand regions in the Aβ(1–40) fibrils (mainly around residues 10–22 and 30–38), which are connected by a short hairpin so that U-shaped monomers form the fibrils, while the N-terminus of the peptide is unstructured and more dynamic^15^,^16^,^17^. Here, we report data on the secondary structure and tertiary interresidual contacts of mature Aβ fibrils grown from pGlu_3_-Aβ(3–40) peptides on the level of selected amino acids.

## Results and Discussions

**Figure 1:**
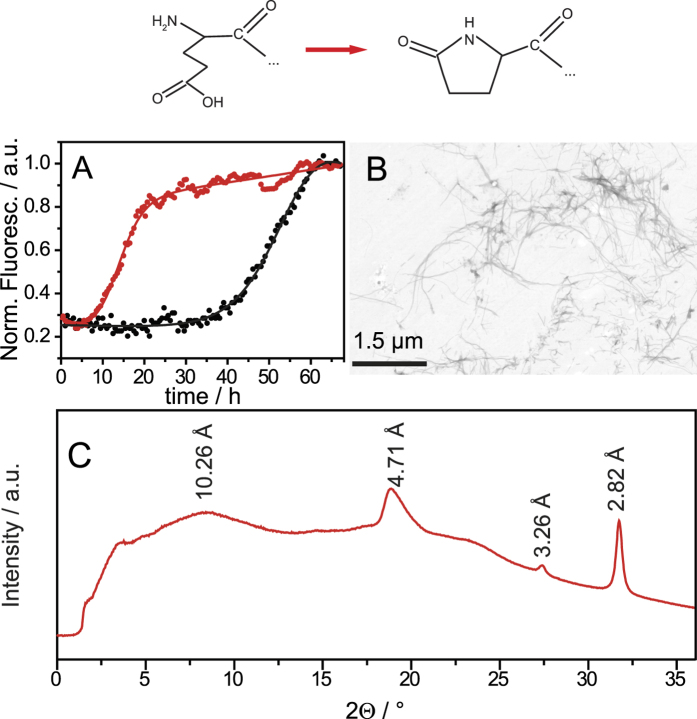
(**A**) ThT fluorescence intensity of pGlu_3_-Aβ(3–40) (red) and Aβ(1–40) (black) as a function of time. (**B**) Scanning transmission electron micrograph of the pGlu_3_-Aβ(3–40) fibrils after 3 weeks of incubation. The scale bar represents 1.5 μm. (**C**) X-ray diffraction pattern of pGlu_3_-Aβ(3–40) fibrils. Above, a sketch of the structural modification from glutamic acid to pyroglutamate is shown.

The fibrillation kinetics of pGlu_3_-Aβ(3–40) in comparison to WT Aβ(1–40) was monitored by standard thioflavin T (ThT) fluorescence spectroscopy. Figure 1A shows the maximum of the ThT fluorescence intensity as a function of time. In agreement with previous results^6^, it is observed that also under our conditions the fibrillation of pGlu_3_-Aβ(3–40) has a significantly shorter lag time and is overall faster than for WT Aβ(1–40). The lag time for pGlu_3_-Aβ(3–40) is 7 ± 1 h, while it is 43 ± 2 h for Aβ(1–40). The morphology of the pGlu_3_-Aβ(3–40) fibrils was studied by electron microscopy (EM). Figure 1B shows a typical EM micrograph of pGlu_3_-Aβ(3–40) fibrils after 3 weeks of incubation, displaying fibrils of homogeneous morphology. These fibrils have a width of 12.8 ± 2.1 nm (n = 20), which is slightly larger than the mean diameter of WT Aβ(1–40) fibrils (10.0 ± 1.6 nm)^18^. A preferential shorter fibril length for pGlu_3_-Aβ(3–40) as reported in^6^ could not be observed under our fibrillation conditions. Fibrils of pGlu_3_-Aβ(3–40) exhibit a mean length of 850 ± 300 nm WT Aβ(1–40) fibrils of 500 ± 200 nm (n = 30). Also, the X-ray diffraction pattern of pGlu_3_-Aβ(3–40) fibrils (Fig. 1C) exhibit the typical cross-β structure as observed for all other amyloid fibrils. The measured main X-ray reflections correspond to repeat spacings of 4.7 Å and 10.3 Å, which represent the typical values for the interstrand spacing and the intersheet distance, respectively^19^.

To obtain insights into the secondary structure on the level of individual amino acids, solid-state NMR spectra of pGlu_3_-Aβ(3–40) fibrils with uniformly ^13^C/^15^N-labeled amino acids (for the labeling scheme see experimental section) were measured. In choosing the sites for isotopic labeling, we paid special attention to the N-terminus of pGlu_3_-Aβ(3–40) to study possible differences in the structures due to the pGlu modification in position 3 and to probe the extent of the known secondary structure elements. To assign the ^13^C and ^15^N chemical shifts, ^13^C-^13^C DARR and ^15^N-^13^Cα NMR correlation spectra were conducted under magic-angle spinning (MAS) conditions in dual acquisition mode^20^. Supplementary Figure S1 shows a ^13^C-^13^C DARR and a ^15^N-^13^Cα NMR spectrum as examples. The chemical shifts values for all labeled amino acids are listed in Table S1.

**Figure 2:**
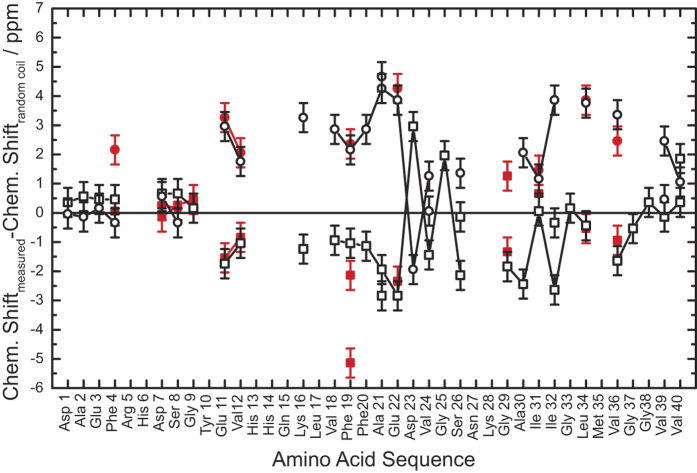
Comparison of the secondary ^13^C MAS NMR chemical shifts of pGlu_3_-Aβ(3–40) (red) and mature WT Aβ(1–40) (black) fibrils for ^13^Cα (squares) and ^13^Cβ (circles). Data are given as the difference of a measured chemical shift to random coil chemical shifts taken from the literature^22^.

Figure 2 reports the ^13^Cα and ^13^Cβ chemical shifts of pGlu_3_-Aβ(3–40) and mature WT Aβ(1–40) fibrils (data taken from ref. 21) as differences from random coil values reported in the literature^22^. Since NMR chemical shifts are sensitive to the secondary structure, values close to zero correspond to random coil regions, while negative values for Cα and positive values for Cβ report β-strand conformations^22^. One can clearly see that most of the chemical shift values of both fibrillar species are very similar. Some alterations are observed for the Cβ signal of Phe_4_, which may result from the direct vicinity to the chemically modified pGlu_3_. For Phe_19_ Cβ and Gly_29_ Cα, two chemical shift values were observed. Such structural polymorphism of Aβ fibrils has been observed before in several preparations of WT Aβ fibrils^15^,^23^,^24^,^25^. Also the larger line width up to 3 ppm (FWHM) for some signals (e.q. Val_12_ or Val_36_) could be a result of structural polymorphism.

Overall, a striking structural similarity between pGlu_3_-Aβ(3–40) and WT Aβ(1–40) fibrils is observed and one has to conclude that the typical secondary structure elements of WT Aβ(1–40) with an unstructured N-terminus and two β-strand regions comprising amino acids 10–22 and 30–38, which are connect by a short unstructured region, holds also true for pGlu_3_-Aβ(3–40).

**Figure 3:**
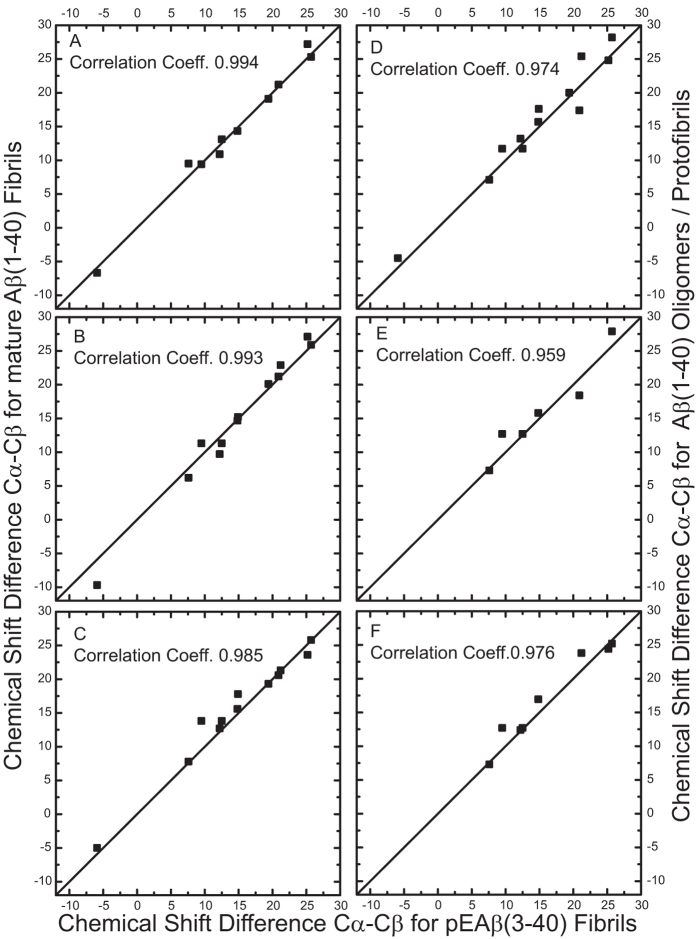
Comparison of fibrils of pGlu_3_-Aβ(3–40) with published chemical shift data of WT Aβ preparations. Correlation plots of the chemical shift differences (^13^Cα-^13^Cβ) for all labeled amino acids of pGlu_3_-Aβ(3–40) (x axis) and Aβ fibrils in different stages of the fibrillation process (y axis) are given. (**A**) mature fibrils from^24^, (**B**) mature fibrils^16^, (**C**) mature fibrils^21^, (**D**) protofibrils^35^, (**E**) oligomers^43^, and (**F**) oligomers^44^. The Pearson correlation coefficient is given for each plot. Note that Gly_9_ and Gly_29_ are not part of this comparison as ^13^Cα-^13^Cβ chemical shift differences are analyzed.

For a systematic comparison of the secondary structure of pGlu_3_-Aβ(3–40) fibrils to different preparations of WT Aβ fibrils in different stages of the fibrillation process, Figure 3 shows correlation plots of the differences in the chemical shift values (^13^Cα-^13^Cβ), which are very sensitive to secondary structure and have the advantage of being independent of chemical shift referencing, which may vary between the different laboratories. On the secondary structure level, a very good correlation of the pGlu_3_-Aβ(3–40) fibrils with the results for WT Aβ fibrils is obtained (A–C). Only slightly smaller correlation coefficients are obtained when pGlu_3_-Aβ(3–40) fibrils are compared to protofibrils (D) and oligomers (E, F). This indicates that the secondary structure of the pGlu_3_-Aβ(3–40) fibrils is very similar to mature fibrils, but also to oligomers and protofibrils.

Tertiary structure information for Aβ(1–40) fibrils represents a field of some controversy and is highly dependent on the number and precision of the structural constraints available, which has led to different structural models^16^,^17^,^26^,^27^. To obtain some insight into the spatial relationship of the two β-strands and the tertiary structure of the pGlu_3_-Aβ(3–40) peptides in the fibrils, ^13^C-^13^C DARR NMR experiments were conducted with a long mixing time of 500 ms. This allows to observe interresidual contacts between carbons in spatial proximity of up to ~6 Å, via the detection of cross peaks between carbons of different amino acids. For mature Aβ(1–40) fibrils as well as oligomers and protofibrils, a contact between the side chains of Phe_19_ and Leu_34_ has been well-described^16^,^17^,^18^,^28^,^29^. This contact indicates the close proximity between the two β-strands of the monomer and the U-shaped structure of the monomers in the fibrils. In the DARR NMR spectrum for pGlu_3_-Aβ(3–40) fibrils (Figure S1), cross peaks between Phe_19_ and Leu_34_ are clearly visible, especially between the aromatic ring carbons of Phe_19_ and the Cβ signal of Leu_34_. This suggests a close structural relationship between pGlu_3_-Aβ(3–40) and WT Aβ(1–40) fibrils also on the tertiary structure level.

It was shown that a molecular contact between Glu_22_ and Ile_31_ indicates earlier stages of the fibrillation process in oligomers and protofibrils^30^,^31^,^32^, but this contact is absent in mature Aβ fibrils^32^. Based on these observations and other data, a model for the reorganization of the hydrogen bonds from intramolecular for oligomers and protofibrils to intermolecular hydrogen bonds for the mature fibrils has been proposed^30^,^31^. In our ^13^C-^13^C DARR NMR spectrum of the peptide that contains these two amino acids ^13^C/^15^N-labeled, such a cross peak indicative of the molecular contact was not observed (Figure S2). This result also confirms that fibrils of pGlu_3_-Aβ(3–40) exhibit a strong structural similarity to mature Aβ(1–40) fibrils.

**Figure 4:**
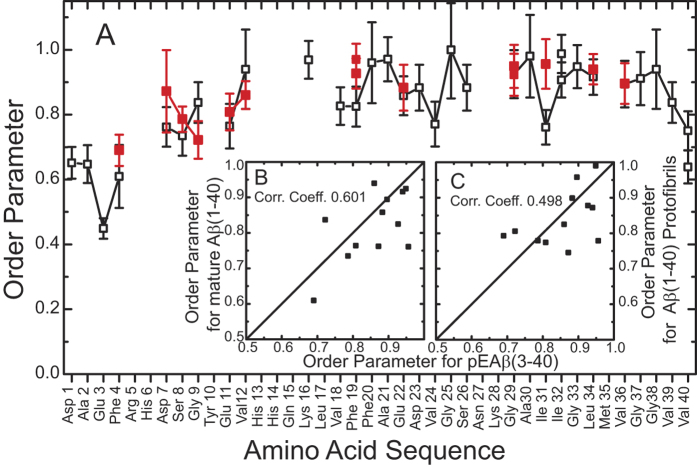
(**A**) Comparison of the ^13^Cα-^1^H order parameters of pGlu_3_-Aβ(3–40) (red) and mature Aβ(1–40) (black) fibrils^21^. Correlation plots of the ^13^Cα-^1^H order parameters of pGlu_3_-Aβ(3–40) with (**B**) mature Aβ(1–40)^21^ and (**C**) with Aβ(1–40) protofibrils^35^ are shown as insets. The Pearson correlation coefficient is given for both correlation plots.

Solid-state NMR also offers the possibility to investigate the molecular dynamics of the individual segments of the pGlu_3_-Aβ(3–40) fibrils. Such measurements can provide information about the different domains in fibrillar peptides or proteins and substantially support the structural data^21^,^33^,^34^. We measured the motionally averaged ^1^H-^13^C dipolar couplings for each resolved carbon signal in DIPSHIFT experiments and convert these into molecular order parameters. Figure 4 shows the comparison of the order parameters of the backbone Cα of pGlu_3_-Aβ(3–40) and Aβ(1–40) (data from ref. 21). For most of the investigated amino acids, the values are similar within the error of the measurement. The deviations for Phe_4_ and Ser_8_ may be explained by the close proximity to the pyroglutamyl-modified N-terminus, which likely alters the dynamics of this unstructured part of the fibrils. This results in a somewhat higher order parameter, which corresponds to smaller motional amplitudes of the fluctuations of these residues. One further notable exception is the order parameter of Ile_31_, which is significantly higher in pGlu_3_-Aβ(3–40) compared to WT Aβ(1–40). This may suggest some importance of this residue for the formation of oligomers and protofibrils as Ile_31_ appears to be involved in intramolecular contacts of intermediates, but not in mature fibrils. Interestingly, the correlations of the measured order parameters to the only available datasets for mature Aβ(1–40) fibrils^21^ and Aβ(1–40) protofibrils^35^ are not as good as observed for the chemical shifts/secondary structure. This may in part reflect the fact that chemical shifts are measured more precisely than motionally averaged dipolar couplings, but could also indicate a dynamic polymorphism that relates to small packing differences of the individual residues in the fibrils.

## Conclusion

Overall, we conclude that on the level of the single amino acid, fibrils formed of pGlu_3_-Aβ(3–40) exhibit a strong similarity in the molecular structure compared to WT mature Aβ fibrils. Our data agree with a recent study that reported, on the basis of H/D exchange NMR, FTIR, and CD measurements that modified pGlu_3_-Aβ(3–40) and unmodified Aβ(1–40) comprised similar peptide conformations^6^. In this study, fibrils of pGlu_3_-Aβ(3–40) of shorter length have been reported, which was not confirmed by our EM data. Although the pGlu modification on the N-terminus of truncated Aβ peptides significantly accelerates the fibrillation, the end product of this structure forming process shows an astonishing similarity to the well described structural features of all mature Aβ fibrils^15^,^16^,^23^. This suggests once more that the physiological effects of the pGlu peptides must be mediated by transient oligomers, which are very difficult to characterize. However, the N-terminus of the pGlu_3_-Aβ(3–40) fibrils showed dynamical alterations, that may have an effect of the stability of the intermediates as well as the fibrils as speculated before^6^.

## Methods

### Sample preparation

Three pGlu_3_-Aβ(3–40) peptides with uniformly ^13^C/^15^N-labeled amino acids in different positions were synthesized using standard Fmoc protocols. The labeling schemes are as follows. Peptide I: Ser_8_, Val_12_ Phe_19_, and Leu_34_ labeled; Pepitde II: Phe_4_, Glu_11_, Gly_29_, and Val_36_ labeled; and Peptide III: Asp_7_, Gly_9_, Glu_22_, and Ile_31_ labeled. The peptides were solubilized in 50 mM Tris buffer (100 mM NaCl, 0.01% NaN_3_, pH 8) at a concentration of 0.1 mg/ml. For fibrillation, the peptide solutions were incubated at 37 °C and shaken at 230 rpm for 3 weeks.

### ThT fluorescence measurements

The fibrillation kinetics of pGlu_3_-Aβ(3–40) was followed by ThT fluorescence intensity measurements. Buffer conditions for fibrillation were the same as above with additional 20 μM ThT in the incubation solution. Volumes of 150 μl were pipetted into the wells of a 96-well plate, which was placed in a Tecan infinite M200 microplate reader (Tecan Group AG, Männedorf, Switzerland). The temperature was kept at 37 °C and a kinetic cycle was applied, such that a 2 s shaking time (2 mm shaking amplitude) followed by a 5 min waiting time was repeated four times with one additional 2 s shaking at the end and the subsequent fluorescence measurement. Fluorescence excitation was set to 440 nm and emission was measured at 482 nm. The fluorescence intensity was measured in increments of 30 min for an overall time period of 65 h. For comparison, also the kinetics of WT Aβ(1–40) fibrillation was recorded under the same conditions. Data was analyzed using procedures reported in the literature^36^.

### Electron microscopy

The fibril morphology was checked by electron microscopy (EM). Fibril solutions were diluted 1:1 with pure water and 1 μl droplets of this solution were applied on formvar coated copper grids, allowed to dry for about 1 h and negatively stained with 1% uranyl acetate in pure water. Scanning transmission electron micrographs were recorded using Zeiss SIGMA, (Zeiss NTS, Oberkochen, Germany) equipped with a STEM detector and Atlas Software.

### Solid-state MAS NMR spectroscopy

For NMR measurements, fibril solutions were ultracentrifuged at ~200,000× g for 4 h at 4 °C. The pellets were lyophilized, rehydrated to 50 wt% H_2_O, homogenized by several freeze-thaw cycles and finally transferred into 3.2 mm MAS rotors. All MAS NMR experiments were conducted on a Bruker 600 Avance III NMR spectrometer (Bruker BioSpin GmbH, Rheinstetten, Germany) at a resonance frequency of 600.1 MHz for ^1^H, 150.9 MHz for ^13^C, and 60.8 MHz for ^15^N using a triple channel 3.2 mm MAS probe. Typical pulse lengths were 4 μs for ^1^H and ^13^C and 5 μs for ^15^N. ^1^H-^13^C and ^1^H-^15^N CP contact time were 1 ms at a spin lock field of ~50 kHz. The relaxation delay was 2.5 s. ^1^H dipolar decoupling during acquisition with a radio frequency amplitude of 65 kHz was applied using Spinal64. The MAS frequency was 11,777 Hz. ^13^C chemical shifts were referenced externally relative to TMS.

^13^C-^13^C DARR NMR spectra and ^13^C-^15^N correlation spectra were acquired simultaneously using dual-acquisition^20^. In the same experiment, a two dimensional ^13^C-^13^C DARR NMR spectrum with a mixing time of 500 ms with 128 data points and four identical ^15^N-^13^Cα correlation spectra with 32 data points in the indirect dimensions were measured. The ^15^N-^13^Cα spectra were processed using NMRPIPE software^37^.

To determine ^1^H-^13^C dipolar couplings, constant time DIPSHIFT experiments^38^ were performed. For homonuclear decoupling during dipolar evolution a frequency switched Lee-Goldburg (FSLG)^39^ with an effective radio frequency field of 80 kHz was used. The MAS frequency for DIPHSIFT experiments was 5 kHz. After Fourier transformation in the direct dimension the signal intensities of the dephasing curve for each resolved carbon was simulated and the determined coupling was divided by the known rigid limit values to obtain the order parameters^40^,^41^. The temperature for all NMR experiments was 30 °C.

### X-ray diffraction measurements

For X-ray diffraction measurements, fibril samples from the MAS rotors were placed on nylon loops (Hampton Research, Aliso Viejo, CA, USA) and mounted onto the goniometer head of a X-ray source (Rigaku copper rotating anode MM007 with 0.8 kW, Tokyo, Japan). The signals were recorded using an image plate detector (Rigaku, Tokyo, Japan) with an exposure time of 180 s at room temperature. Diffraction images were analyzed using ImageJ^42^.

## Additional Information

**How to cite this article**: Scheidt, H. A. *et al*. Fibrils of Truncated Pyroglutamyl-Modified Aβ Peptide Exhibit a Similar Structure as Wildtype Mature Aβ Fibrils. *Sci. Rep.* **6**, 33531; doi: 10.1038/srep33531 (2016).

## Supplementary Material

Supplementary Information
