# Supplementary material for: Fibrils of Truncated Pyroglutamyl-Modified Aβ Peptide Exhibit a Similar Structure as Wildtype Mature Aβ Fibrils
Source: Sci Rep. 2016 Sep 21;6:33531. doi: 10.1038/srep33531 (PMC5030707; doi:10.1038/srep33531)
Supplement: Supplementary Information [file srep33531-s1.pdf]

Supporting information

Fibrils of Truncated Pyroglutamyl-Modified Aβ Peptide Exhibit a Similar Structure as Wildtype Mature Aβ Fibrils

Holger A. Scheidt[a],*, Juliane Adler[a], Martin Krueger[a], Daniel Huster[a],*

Table S1 Isotropic chemical shifts (relative to TMS) for pE-Aβ fibrils (for ^{13}C relative to TMS)

Amino Acid	Cα/ ppm	Cβ/ ppm	C=O/ ppm	Cγ/ ppm	Cδ/ ppm	N/ ppm
Phe 4	55.2	40.3	n.d.			120.4
Asp 7	51.6	39.4	n.d.	178.0		120.0
Ser 8	56.3	62.2	173.0			124.8
Gly 9	43.6		170.6			109.8
Glu 11	52.7	31.5	172.5	34.5	181	126.0
Val 12	58.5	32.8	172.1	19.2		123.1
Phe 19	50.0/53.0	40.5	172.5			125.2
Glu 22	51.9	32.5	173.8	34.3	180.7	120.2
Gly 29	41.8/44.3		170.2			109.3
Ile 31	59.0	38.1	172.2	25.9/17.1	11.7	124.9
Leu 34	51.9	44.3	171.8	25.5	23.5	115.4
Val 36	58.4	33.2	173.4	21.9/19.4		124.7

[1] two signals observed, strongest signal listed first, n.d.- not detected

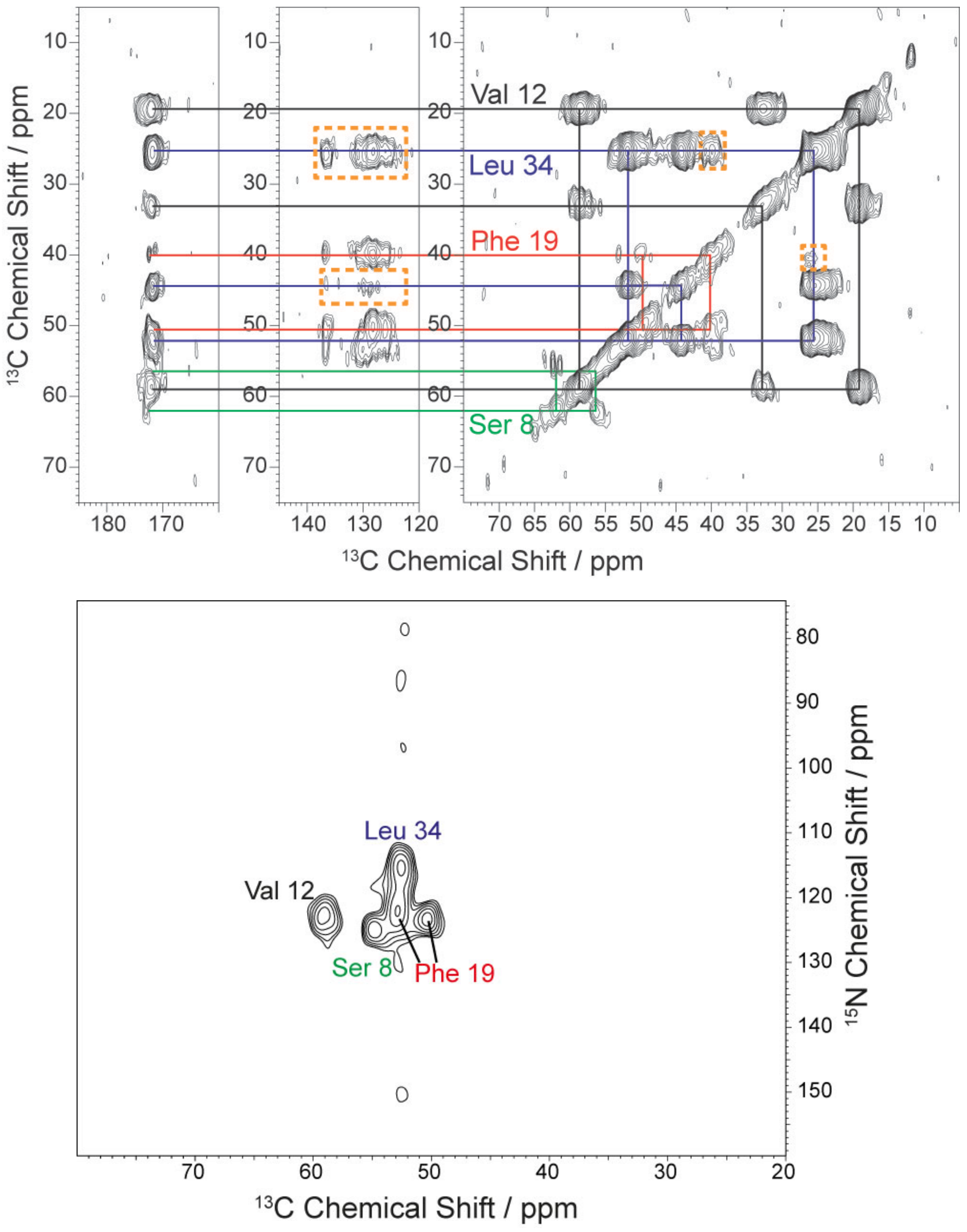

Sup Figure 1: ^{13}C-^{13}C DARR (above) and ^{15}N - ^{13}Cα (below) NMR spectra of pE3-Aβ(3-40) peptide I fibrils with a DARR mixing time of 500 ms at a MAS frequency of 11777 Hz and a temperature of 30°C. The assignment for the labeled amino acids is shown Ser 8 (green), Val 12 (black), Phe 19 (red) and Leu 34 (blue). The orange boxes in the DARR spectrum indicate interresidual crosspeaks between Phe 19 and Leu 34.

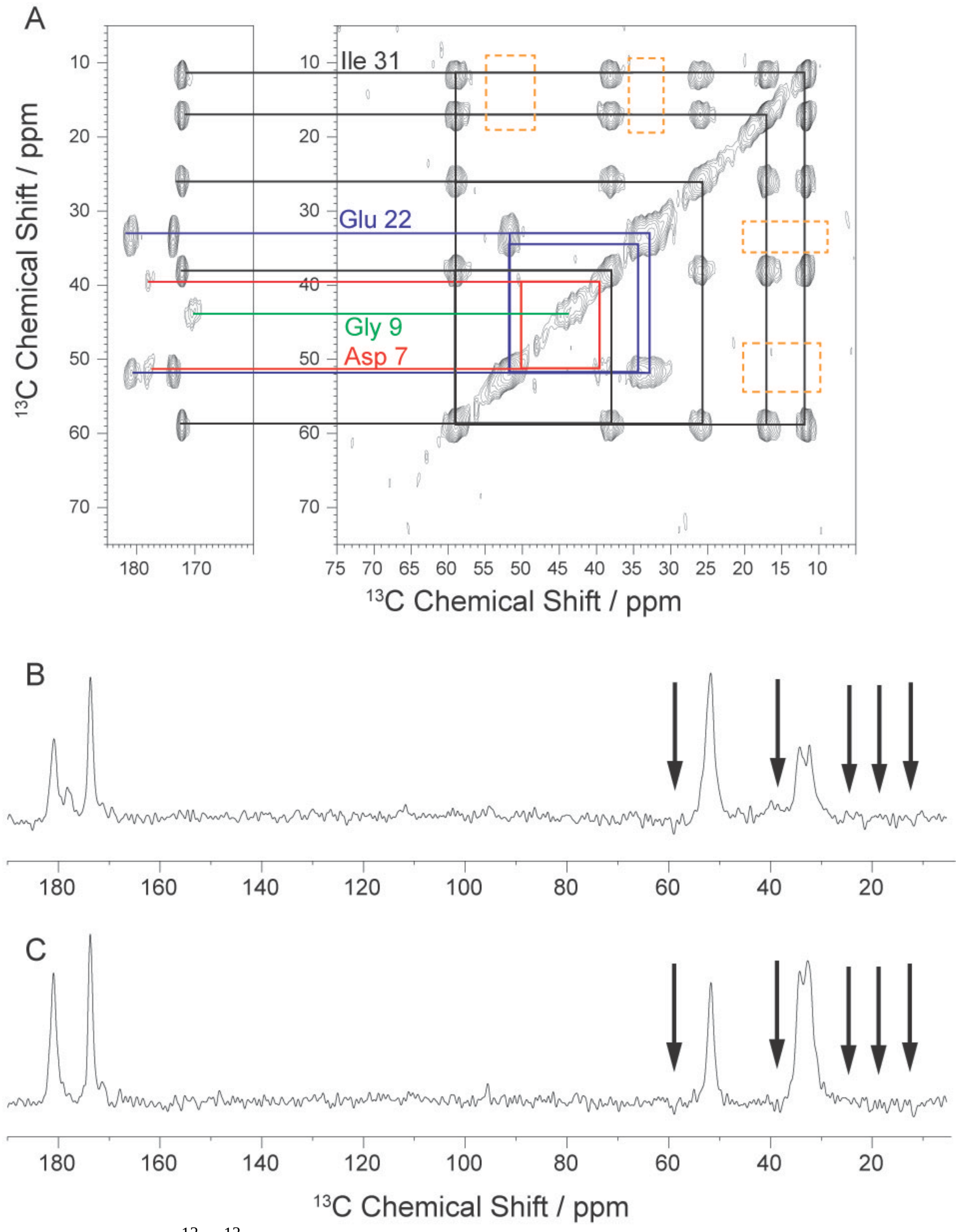

Figure S2: (A) ^{13}C-^{13}C DARR MAS NMR spectrum of pGlu$_3$-Aβ(3-40) peptide fibrils recorded at a mixing time of 500 ms, a MAS frequency of 11,777 Hz and a temperature of 30°C. The assignment for the labeled amino acids is shown Asp$_7$ (red), Gly$_9$ (green), Glu$_{22}$ (blue) and Ile$_{31}$ (black). Cross peaks between the side chain of Glu$_{22}$ and Ile$_{31}$ were not observed (orange boxes). Additionally, slices through the Cα (B) and Cβ (C) chemical shifts of Glu$_{22}$ are shown, which indicate that no cross peaks to Ile$_{31}$ are observed (the chemical shifts for the Ile$_{22}$ signals are marked by arrows).
